# Assessment of *Anopheles* salivary antigens as individual exposure biomarkers to species-specific malaria vector bites

**DOI:** 10.1186/1475-2875-11-439

**Published:** 2012-12-31

**Authors:** Zakia M I Ali, Mahfoud Bakli, Albin Fontaine, Nawal Bakkali, Vinh Vu Hai, Stephane Audebert, Yvan Boublik, Frederic Pagès, Franck Remoué, Christophe Rogier, Christophe Fraisier, Lionel Almeras

**Affiliations:** 1 Unité de recherche en biologie et épidémiologie parasitaires (URBEP) – UMR63 – IFR48, Institut de Recherche Biomédicale des Armées (Armed forces biomedical research institute, IRBA), antenne Marseille, GSBdD de Marseille Aubagne, 111 avenue de la corse, BP 40026, Marseille cedex 02, 13568, France; 2 Aix Marseille Université, Unité de Recherche en Maladies Infectieuses et Tropicales Emergentes (URMITE), UM63, CNRS 7278, IRD 198, Inserm 1095, WHO collaborative center for rickettsioses and other arthropod borne bacterial diseases, Faculté de Médecine, 27 bd Jean Moulin, Marseille cedex 5, 13385, France; 3 Marseille Proteomic (MaP), Centre de Recherche en Cancérologie de Marseille (CRCM), INSERM CNRS, Aix-Marseille Université, Institut Paoli-Calmette, 27, boulevard Leï Roure, BP 30059, Marseille cedex 09, 13273, France; 4 CNRS/CRBM, UMR5237, Montpellier, F-34293, France; 5 Institut de Recherche Biomédicale des Armées (IRBA), antenne Marseille, Unité d’Entomologie Médicale, URMITE UMR 6236, GSBdD de Marseille Aubagne, 111 avenue de la corse, BP 40026, Marseille cedex 02, 13568, France; 6 Cire Océan indien, 2bis avenue Georges Brassens, Saint Denis Cedex 9, CS 60050-97408, LA REUNION; 7 Laboratoire Maladies Infectieuses et Vecteurs: Ecologie, Génétique, Evolution et Contrôle, UMR 224 CNRS/IRD/UM1, Montpellier, France; 8 Institut Pasteur de Madagascar, Ambohitrakely, 101, B.P. 1274, Antananarivo, Madagascar

**Keywords:** *Anopheles gambiae*, *Anopheles funestus*, Antigenic salivary proteins, SG6, 5′nucleotidase, Exposure biomarkers, Epidemiology

## Abstract

**Background:**

Malaria transmission occurs during the blood feeding of infected anopheline mosquitoes concomitant with a saliva injection into the vertebrate host. In sub-Saharan Africa, most malaria transmission is due to *Anopheles funestus s.s* and to *Anopheles gambiae s.l.* (mainly *Anopheles gambiae s.s.* and *Anopheles arabiensis*). Several studies have demonstrated that the immune response against salivary antigens could be used to evaluate individual exposure to mosquito bites. The aim of this study was to assess the use of secreted salivary proteins as specific biomarkers of exposure to *An*. *gambiae* and/or *An*. *funestus* bites.

**Methods:**

For this purpose, salivary gland proteins 6 (SG6) and 5′nucleotidases (5′nuc) from *An. gambiae* (gSG6 and g-5′nuc) and *An. funestus* (fSG6 and f-5′nuc) were selected and produced in recombinant form. The specificity of the IgG response against these salivary proteins was tested using an ELISA with sera from individuals living in three Senegalese villages (NDiop, n = 50; Dielmo, n = 38; and Diama, n = 46) that had been exposed to distinct densities and proportions of the *Anopheles* species. Individuals who had not been exposed to these tropical mosquitoes were used as controls (Marseille, n = 45).

**Results:**

The IgG responses against SG6 recombinant proteins from these two *Anopheles* species and against g-5′nucleotidase from *An*. *gambiae*, were significantly higher in Senegalese individuals compared with controls who were not exposed to specific *Anopheles* species. Conversely, an association was observed between the level of *An. funestus* exposure and the serological immune response levels against the f-5′nucleotidase protein*.*

**Conclusion:**

This study revealed an *Anopheles* salivary antigenic protein that could be considered to be a promising antigenic marker to distinguish malaria vector exposure at the species level. The epidemiological interest of such species-specific antigenic markers is discussed.

## Background

Malaria remains a major public health burden, affecting approximately 240 million individuals annually and causing more than 800,000 deaths, mainly in populations living in tropical and sub-tropical countries in sub-Saharan Africa [1]. To date, the absence of a licensed malaria vaccine [2-4] and the spread of parasite resistance against malaria treatment [5] necessitate strengthening the control of malaria exposure by avoiding host/vector contact. Thus, several strategies could be used to protect individuals from mosquito bites, either by using personal anti-vectorial devices, like impregnated bed nets, repellents, and long-sleeved clothes [6], or by controlling vector populations at both the adult and larval stages [7,8]. The effectiveness of these anti-vectorial devices is generally evaluated with parasitological and entomological methods [9-11]. Although these methods have demonstrated their capacity to estimate human exposure to malaria vectors and *Anopheles* densities, these tools lack important logistics and present limited efficiency in the context of low-level exposure to *Anopheles* bites. In addition, they are not designed for the assessment of the heterogeneity of mosquito exposure at the individual level [9]. Therefore, the development of new indicators and methods to evaluate the effectiveness of anti-vectorial strategies at the individual level is necessary.

Mosquito salivary proteins injected into the host during blood feeding play a dual role by counteracting homeostasis and modulating the vertebrate immune response [12]. In addition to their role in the blood meal, some salivary proteins presenting immunogenic properties could elicit an antibody response by their host. This immune response, initially described in allergic individuals [13], has been proposed as a marker of exposure to mosquito bites [14,15]. Thus far, several studies have demonstrated that the level of IgG immune responses against salivary antigens is associated with the level of individual exposure to mosquito bites, which may vary according to seasonal mosquito density [15,16], transient exposure following travel in malaria-endemic areas [17] or following the introduction of anti-vectorial measures, such as the use of insecticide-treated nets [18]. However, the existence of homologous salivary protein sequences that are shared among different species from *Culicidae* requires the identification of specific antigenic proteins or peptides prior to developing any anti-saliva based immunological tools to assess individual exposure to different mosquito vectors [9,19].

Among mosquito salivary proteins, the *Anopheles gambiae* salivary gland protein 6 (gSG6) was proposed as a potential candidate for the examination of specific malaria vector exposure markers [20]. This small protein, expressed specifically in the salivary glands of adult female mosquitoes, was selected based on its restrictive presence in species belonging to the subgenus *Cellia*, including major Afrotropical malaria vectors (e g, *An. gambiae* species complex, *Anopheles funestus*) [21], and its immune recognition by individuals exposed to *Anopheles*[17]. To limit production costs, Poinsignon and colleagues designed a gSG6-based peptide sequence (gSG6-P1) according to its predicted immunogenic properties [20]. The gSG6-P1 peptide was repeatedly reported to be a relevant *An. gambiae*-specific marker of exposure [20-23]. Moreover, the high level of amino-acid conservation between gSG6-P1 peptide sequences from *An.gambiae* and *An. funestus* indicate the potential of this peptide to be an indicator of exposure to both of these main vectors of *Plasmodium falciparum* in Africa [24]. Similar observations were obtained using recombinant forms of whole SG6 orthologs from *An. gambiae* and *An. funestus*[25,26], which could be attributed to the high level of identity among them (i.e., 80%). More recently, it was reported that the level of IgG against gSG6 was positively linked to the risk of malaria pathogen transmission [27]. Thus, SG6 proteins are currently the best and uniquely relevant indicators of exposure to Afrotropical malaria vectors.

However, the identification of salivary antigenic candidates capable of discriminating individual exposure at the species level could improve the development of this type of immunological test by determining the anopheline fauna biting population. Effectively, together with *An. funestus*, mosquitoes from the *An. gambiae s.l.* (*An. gambiae s.s.* and *Anopheles arabiensis*) are the most common vectors of human malaria in sub-Saharan Africa [28]. These highly anthropophilic *Anopheles* species could geographically co-inhabit most sub-Saharan countries [29]. Malaria parasites can thus be transmitted by multiple and often sympatric vectors [30-32]. However, anopheline fauna could be spatially and temporally influenced by several factors, such as environmental conditions that could seasonally modify the anopheline species proportions and densities. During the dry season, the *An. gambiae s.l*. density is decreased for the benefit of *An. funestus*. The maintenance of malaria transmission at several sites could be attributed to the presence of *An. funestus*[33]. Thus, in addition to the use of salivary exposure markers to estimate the individual level of exposure to Afrotropical malaria vectors, the characterization of new species-specific anopheline salivary antigenic candidates could be useful for determining the predominant mosquito populations of a study area. Such information could be useful for the adaptation of vectorial control measures against specific mosquito populations or for the estimation of the risk of malaria transmission or persistence.

Thus, the aim of this study was to assess anopheline salivary proteins that could be used as species-specific exposure biomarkers to distinguish *An*. *funestus* exposure from *An*. *gambiae s.l.* exposure. First, SG6 salivary proteins from *An*. *gambiae s.s.* (gSG6) and *An*. *funestus* (fSG6) were produced in recombinant forms and evaluated on sera from individuals that were either un-exposed to *Anopheles* or exposed predominantly to *An*. *funestus* or *An*. *gambiae s.l.*, to confirm that these salivary proteins could be used to detect a predominant exposure to either of these two mosquito species. In addition, recombinant forms of 5′nucleotidase salivary proteins from *An*. *gambiae s.s.* (g-5′nuc) and *An*. *funestus* (f-5′nuc) were tested on the same sera to assess their potential as species-specific indicators of exposure. The specificity of the IgG response against these selected salivary proteins at the genus or species levels was analyzed by ELISA using sera from individuals living in three Senegalese villages (NDiop, n = 50; Dielmo, n = 38; and Diama, n = 46) exposed to distinct densities and proportions of the *Anopheles* species. Individuals that were not exposed to these tropical mosquitoes were used as controls (Marseille, n = 45).

## M**ethods**

### Ethics statement

The protocol (N°2006-A00581-50) was approved by the Marseille-2 Ethical Committee (France) and by the Senegal National Ethics Committee (Dakar, Senegal). The written informed consent of each participant was obtained at the beginning of the study, after a thorough explanation of its purpose.

### Study sites, sera samples and entomological observations

The study was conducted on two different populations: un-exposed people and people who were regularly exposed to *An. gambiae s.l.* and *An. funestus* bites*.* Forty-five serum samples from French adults living in Marseille (43°17′N, 5°22′E; mean age ± standard deviation (SD): 40.73 ± 12.02, sampled in February 2007 [16]), who had never been in countries endemic for *An. gambiae s.l.* and *An. funestus*, were used as un-exposed negative controls. The exposed group consisted of 134 individuals living in the Senegalese villages of Diama (16°13′ N, 16°23′W; n = 46, mean age ± SD: 17.96 ± 11.58, sampled between March and October 1994), Dielmo (13°45′N, 16°25′W; n = 38, 28.38 ± 21.26, sampled in March 1995) and Ndiop (13°14′N, 16°23′W; n = 50, 25.87 ± 18.34, sampled between March and December 1995). These populations were exposed to high (Dielmo, approximately a 30.6 human biting rate (HBR), moderate (Ndiop, approximately a 3.9 HBR) and low (Diama, <1 HBR) *Anopheles* bite levels. Individuals living in Dielmo and Ndiop were predominantly exposed to *An. funestus* (approximately 66%) and *An. gambiae s.l.* (approximately 95%), respectively. Individuals living in Diama were predominantly exposed to other anopheline mosquito species (*Anopheles pharoensis*, approximately 87%). Details regarding the entomological data are presented in Table 1 and show for each site the amount and the proportion of each anopheline species collected from the three months preceding blood sampling until the end of the blood sampling period. Concerning entomological measures, adult mosquitoes were collected monthly using human bait catches and the HBR, which is the number of mosquito bites per person per night, was calculated as the number of mosquitoes captured during the month divided by the number of person-nights. Additional data on the study site, including entomological and parasitological factors, have been previously reported [34-40].

**Table 1:** **Characteristics of density and exposure to*Anopheles*bites in each site according to entomological data**

**Location**	**Number of*Anopheles*caught**	**Percentage composition**	**HBR (An./person/night)**	**Collection period**	**References**
**Diama**	**1,492**		**<1**	January '94 – December '94	[37]**,**[38]**,**[39]
*An. gambiae s.l.*	17	1.1%			
*An. gambiae s.s.*	n.d.	n.d.			
*An. arabiensis*	n.d.	n.d.			
*An. funestus*	0	0.0%			
Others Anophelines (86.9% of *An. pharoensis*)	1475	98.9%			
**Dielmo**	**1,473**		**30.6**	January '95 to march '95	[34]
*An. gambiae s.l.*	494	33.5%	10.3		
*An. gambiae s.s.*	13	0.9%	0.3		
*An. arabiensis*	481	32.6%	10.0		
*An. funestus*	978	66.4%	20.3		
Others Anophelines	1	0.0%	0.0		
**NDiop**	**597**		**3.9**	January '95 – December '95	[35]**,**[36]
*An. gambiae s.l.*	565	94.6%	3.7		
*An. gambiae s.s.*	190	32%	1.3		
*An. arabiensis*	373	63%	2.5		
*An. funestus*	28	4.7%	0.2		
Others Anophelines	4	0.7%	0.0		

n.d.: not determined, HBR: human biting rate.

### Protein expression and purification

The coding sequences for the anopheline proteins gSG6 (gi|13537666), g-5′nucleotidase (gi|4582528), fSG6 (gi|114864550) and f-5′nucleotidase (gi|114864746) were retrieved from the National Center for Biotechnology Information (NCBI) database. The cDNAs of selected proteins were synthesized with a C-terminal His-tag and cloned into the baculovirus expression vector pFast Bac1 (Invitrogen, Cergy Pontoise, France) by Genecust (Gencust, Dudelange, Luxembourg). The fidelity of the cloned sequences was verified by DNA sequencing, using an ABI Prism 3100 analyzer (Applied Biosystems). Recombinant bacmid DNA was generated in the DH10Bac *Escherichia coli* strain (Invitrogen), using the Bac-to-Bac system and protocol (Invitrogen). *Spodoptera frugiperda* (Sf9) cells were transfected with the recombinant bacmid DNA using the Lipofectin transfection reagent (Invitrogen), according to the manufacturer’s instructions. The structures of all the inserts were sequenced for authentic cloning (ABI Prism 3100 analyzer, Applied Biosystems). Confirmed clones were amplified in *Spodoptera frugiperda* (Sf9) cells in serum-free medium (Sf-900 II SFM, Gibco, Carlsbad, CA) to produce working viral stocks, which were titrated by a plaque assay and used for subsequent expression studies. For protein production, Sf9 cells were cultured at 28°C in 800 ml of suspension culture (1,3×10^6^ Sf9 cells/ml), infected with a multiplicity of infection of 5 and harvested after three days by centrifugation at 500 × g for 15 min at 4°C, washing in PBS and repeating centrifugation. All pellets were stored at −80°C until use. The cell pellet was resuspended in lysis buffer (50 mM Tris, pH 7.9, 300 mM NaCl, 20 mM imidazole, with proteolysis inhibitor P8849 (Sigma), and disrupted using an Emulsiflex C3 cell disruptor (Avestin, Mannheim, Germany). The cell lysate was clarified by centrifugation at 40,000 rpm for 45 min at 4°C (45 Tirotor, Beckman Coulter). The recombinant proteins were purified using HisTrap HP columns (AKTA purifier 10GEH, GE Healthcare, France). The fractions containing the His-tagged recombinant proteins were selected by SDS-PAGE and pooled. To eliminate contaminant proteins, pooled fractions of each recombinant protein were then purified by gel filtration (superdex 75 26/60 column, GE Healthcare). The fractions containing recombinant proteins were identified by SDS-PAGE, pooled and dialyzed against a 40 mM Tris–HCl buffer (pH 7.5). The protein concentration was measured using a Lowry DC Protein assay (Bio-Rad, Hercules, CA, USA). The purity of purified proteins was determined by SDS-PAGE, and the identity was confirmed by mass spectrometry (MS).

### SDS-PAGE

Five microgram of each purified recombinant protein were reduced in a Tris buffer containing dithiothreitol (1% w/v, Sigma), boiled for 5 min, and loaded per well onto a 15% polyacrylamide gel before to be separated using a Mini PROTEAN II (BioRad, Hercules, CA, USA). After electrophoresis, gels were stained with Coomassie brilliant blue R-250 (Imperial^TM^ Protein Stain, Thermo Scientific, Rockford, IL, USA) and scanned with a high-resolution densitometer scanner (Image Scanner 3, GE Healthcare) and densitometry profiles were analysed using the ImageQuant^TM^ TL software (GE Healthcare). Protein bands from gels were excised for further identification by mass spectrometry. Molecular weights were estimated by comparison with standard molecular weight marker (Biorad).

### In-gel digestion and mass spectrometry analysis

Excised bands were digested overnight at 37°C with sequencing-grade trypsin (12.5 μg/mL; Promega Madison, WI, USA) in 50 mM NH_4_HCO_3_ (Sigma). The resulting peptides were extracted with 25 mM NH_4_HCO_3_ for 15 min, dehydrated with acetonitrile (ACN) (Sigma), incubated with 5% acid formic (Sigma) for 15 min under agitation, then dehydrated with ACN, and finally completely dried using a SpeedVac. The samples were then analysed on a NanoLC-LTQ-OrbitrapVelos-ETD (Thermo Scientific, Bremen, Germany) for identification.

### Enzyme-Linked ImmunoSorbent Assays (ELISA*)*

ELISA was performed according to standard procedures. Microtiter 96-well plates (Nunc Maxisorp Immunoplates, Denmark) were coated for three hours at 37°C with 10 μg/ml (50 μl/well) of either gSG6, g-5′nucleotidase, fSG6 and f-5′nucleotidase purified recombinant anopheline proteins in 0.1M bicarbonate coating buffer (pH 9.6) (Sigma). Three washes were done with 200 μL of PBS (pH 7.4, Sigma, USA) plus 0.05% Tween-20 (Sigma) between each incubation. Plates were blocked overnight at 4°C with 200 μL of blocking buffer consisting of PBS 0.05% Tween and 5% skimmed milk (Beckton, Dickinson Bioscience, USA). Serum diluted 1:50 in blocking buffer was added (50 μl/well) and incubated at 37°C for 1 h. Fifty microliters of horseradish peroxidase (HRP)-conjugated rabbit anti-human IgG (1:10,000, Invitrogen, Rockville, USA) diluted in the blocking buffer were incubated for 1 h at 37°C. Enzyme activity was detected by incubation with 50 μl of tetramethylbenzidine substrate (KPL, USA) for 10 min at room temperature in the dark. The reaction was stopped using 50 μl of 1 M H_2_SO_4_. The optical density (OD) at 450 nm was determined with a microplate reader (Versa Max® Turnable Multiplate Reader, Molecular Devices, UK). Each serum was tested in duplicate against the different recombinant antigenic proteins and also without antigen (coating buffer only). In order to improve result consistencies, sera from different study sites have been randomly loaded on each plate and each individual serum were tested on the same plate against the four recombinant proteins. A pool of eight sera from individuals living in Dielmo and Ndiop sites presenting high level of antibody responses against the four anopheline recombinant proteins tested (selected based on ELISA optimisation tests), were used as a positive control on all plates coated*.* Only plates presenting inter-assay variations in absorbance values of positive controls lower than 20% were included in the analysis. The levels of IgG antibodies were expressed as adjusted OD (aOD), which was calculated for each serum sample duplicate as the mean OD value of antigenic proteins-coated wells minus the mean OD value of the background control wells (*i.e.,* coating buffer without antigenic proteins). Sera whose duplicates showed a coefficient of variation (CV) ≥20% were not included in the analysis. The mean aOD of unexposed individuals plus three standard deviation (SD) was used as cut-off value for seropositivity. Seroprevalence was defined as the pourcentage of seropositive individuals in each group.

### Protein sequence analysis

*Culicidae* protein sequences that were related to the 5′nucleotidases from both *An. funestus* (gi|114864746) and *An. gambiae* (gi|4582528) were retrieved from the National Center for Biotechnology Information (NCBI) using the BLASTp program. The hit sequences exhibiting a significant alignment (E-value<1.10^-4^) and a hit sequence with coverage ≥70% and identity ≥ 50% were selected for further protein sequence comparisons. Multiple sequence alignment was performed with the Clustal W 1.7 multiple sequence alignment program [41], which is included in the Molecular Evolutionary genetic Analysis 5 (MEGA 5) programs package [42].

### Statistical analysis

After verifying that values in each group did not assume a Gaussian distribution, the Kruskal-Wallis test was used for multiple group comparisons and the Friedman test was used to compare observations repeated on the same subjects in each study site. Two independent groups were compared by the Mann–Whitney U test. The Wilcoxon signed-rank test was used for comparison of two paired groups. Frequencies were compared by the Pearson’s Chi-squared test and Spearman’s rank correlation coefficient was computed when appropriate. All differences were considered significant at *p* <0.05 and statistical analysis and figures were performed using the computing environment R (R Development Core Team, 2012).

## Results and discussion

### Degree of conservation of salivary proteins

Members of the 5′nucleotidase/Apyrase family have been ubiquitously found in the saliva of hematophagous arthropods [19]. These enzymes are known to facilitate the acquisition of blood meals by removing and degrading pharmacologically active nucleotides (i.e., ATP/ADP) that are important for platelet aggregation at the site of the injury [43]. Three members of the 5′nucleotidase gene family were described at the transcript level in *An. gambiae* salivary glands [44]. Among them, the putative 5′nucleotidase (gi|4582528) from *An. gambiae* (g-5′nuc) was highly expressed compared to the other two 5′nucleotidase family members. The identification of the putative 5′nucleotidase at the protein level in *An. gambiae* saliva (64 kDa) confirmed that this salivary protein could be injected into the host during blood feeding [45]. Moreover, the induction of host antibody responses against members of the 5′nucleotidase/Apyrase protein family has previously been reported [46-48]. Collectively, these data suggested that 5′nucleotidase proteins could be used as potential immunological exposure markers to mosquito bites. To determine whether members from this protein family could be selected as antigenic candidates for the evaluation of exposure to mosquito bites at the species level, the degree of protein sequence diversity in the 5′nucleotidase/Apyrase protein family among species from *Culicidae* was evaluated.

**Figure 1:**
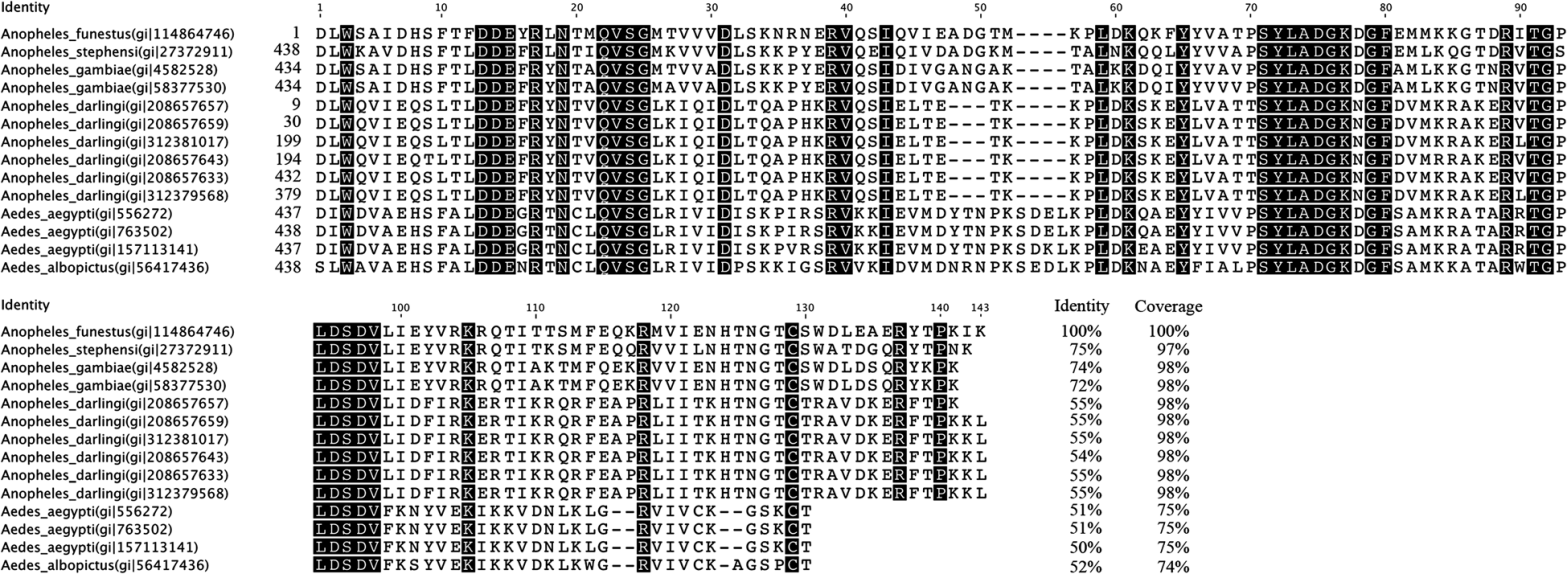
**Alignment of *Culicidae *protein members from the 5′ nucleotidase/Apyrase family.** Using the BLASTp program, 13 *Culicidae* protein sequences related to 5′nucleotidase (gi|114864746) from *An. funestus* were selected (E-value<1.10^-4^, coverage ≥70% and identity ≥ 50%). An alignment of *Culicidae* 5′nucleotidase/apyrase proteins to 5′nucleotidase from *An. funestus* (f5′nuc, gi|114864746) was performed with ClustalW. Only the parts of the protein sequence aligned with the f5′nuc protein sequence are presented. Mosquito species, protein name, accession numbers and amino-acid positions are indicated, and conserved amino acids are shaded. The percentages of identical amino acid residues and sequence recovering compared with f5′nuc (gi|114864746) are listed for each of the selected proteins.

Among 5′nucleotidase anopheline proteins, the 5′nucleotidase from *An. funestus* (f-5′nuc; gi|114864746) was selected based on its protein sequence peculiarities. Indeed, alignment of this sequence with the *An*. *gambiae* putative 5′nucleotidase (g-5′nuc; gi|4582528) indicated that the protein from *An. funestus* lacks a large part of the amino-terminal domain (Additional file 1). The molecular weights and sizes (a.a. length) of f-5′nuc protein sequences (17 kDa, 139 a.a.) differ from those of g-5′nuc (64 kDa, 570 a.a.), which leads to a low coverage sequence (31%) and a moderate identity (<75%). When blasting the f-5′nuc protein sequence (gi|114864746) against the *Culicidae* protein database on NCBInr (NIH, Bethesda), 13 protein hits containing homologous sequences could be retrieved (E-value<1.10^-4^, coverage ≥70% and identity ≥ 50%). An alignment of the f-5′nuc protein sequence (gi|114864746) with the members of the 5′nucleotidase/Apyrase family from other *Culicidae* indicated that orthologous protein sequences from the *Anopheles* and *Aedes* genera presented amino-acid sequence similarities (Figure 1). Among these homologous proteins, two 5′nucleotidase/Apyrase proteins from *An. gambiae* (gi|4582528 and gi|58377530) and one from *Anopheles stephensi* (gi|27372911) presented amino-acid identities between 72-75%, and the identity levels drop to less than 56% for all of the other selected proteins, suggesting an important diversity in this sequence among *Culicidae*. Interestingly, among all the sequences retrieved, only two members of the 5′nucleotidase/Apyrase family (gi|208657657, gi|208657659) from the *Anopheles darlingi* species were also truncated at their amino-terminal end, but these proteins presented moderate identities (<55%) with the 5′nucleotidase from *An. funestus*. Thus, taken together, the f-5′nuc protein peculiarities (*i.e.,* a moderate level of sequence conservation within the *Culicidae* family, an amino-terminal end truncation, the high abundance of these family proteins in the anopheline saliva and their involvement in eliciting host antibody responses) have highlighted our interest in assessing this protein as a potential marker candidate to distinguish exposure to *An*. *funestus* from exposure to *An*. *gambiae s.l.*.

Concerning g5′nuc (gi|4582528), a sequence alignment showed that members of the 5′nucleotidase/Apyrase family share only a moderate level of conservation of the protein sequences (<66% identical amino acids residues), with the exception of a paralogous protein from *An. gambiae* (gi|58377530) and a salivary apyrase from *An. stephensi* (gi|27372911) that possess 99% and 80% identical amino acid residues, respectively (Additional file 2). Due to their determinant role in blood feeding, these enzymes could be submitted to environmental pressures that lead to heterogeneous and independent sequence evolution.

SG6 proteins (gSG6, gi|13537666 and fSG6, gi|114864550) were previously described to be restrictively present in the *Cellia* subgenus and are well conserved within this anopheline subgenus (Additional file 3) [21]. SG6 recombinant proteins were thus used as a reference immunological tool to evaluate the level of exposure to anopheline mosquitoes.

### Production of recombinant SG6 and 5′nucleotidase salivary orthologs from *An. gambiae* and *An. funestus*

**Figure 2:**
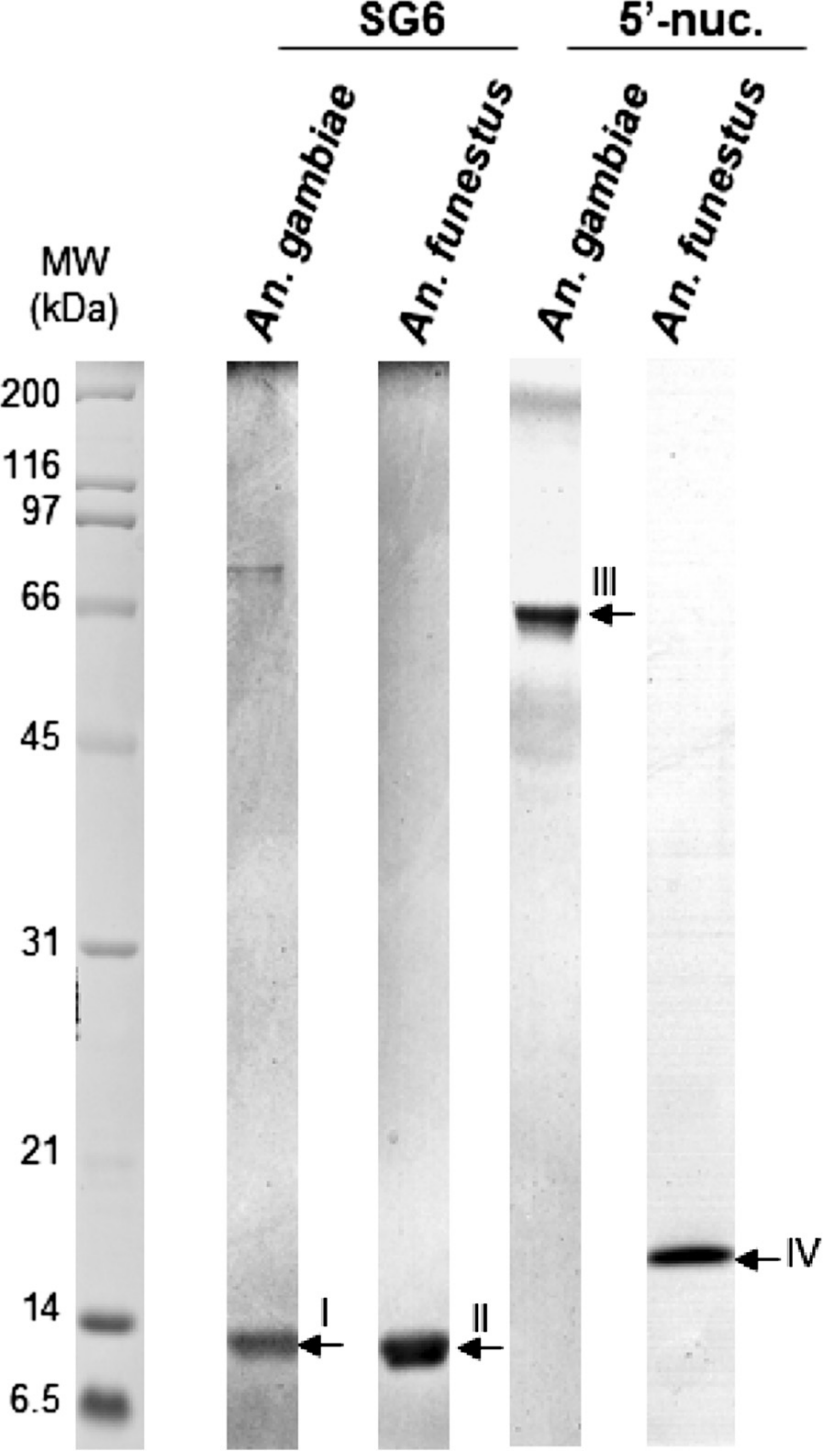
**The assessment of the expression and purification of anophelines salivary proteins.** Recombinant ortholog forms of SG6 and 5′nucleotidases from *An. gambiae* and *An. funestus* expressed in Spodoptera frugiperda (Sf9) cells and purified by affinity and gel filtration chromatography were separated on a 15% SDS-PAGE and post-stained with Imperial™ Protein Stain (Thermo Scientific). Five micrograms of each collected fraction were loaded per well. The protein name and the *Anopheles* species corresponding to each well are indicated at the top of the gel. The Roman numeral numbers on the right side of the gel correspond to the band numbers excised for identification by mass spectrometry. Band identity is listed in Table 2. Standard molecular weights are indicated on the left side. MW: molecular weight. kDa: kilodalton.

A poly-histidine tag was added to the C-terminus of each recombinant protein to ensure the purification of whole recombinant proteins. gSG6 and g-5′nuc salivary proteins from *An. gambiae,* and fSG6 and f-5′nuc salivary proteins from *An. funestus* were produced in Sf9 cells using a baculovirus expression system and were purified by affinity chromatography (HisTrap HP) and a gel filtration column. The fractions corresponding to recombinant proteins were separated by SDS-PAGE, and a representative purified fraction for each protein is presented in Figure 2. As expected, protein bands with apparent molecular weights corresponding to gSG6 and fSG6 protein fractions were detected at approximately 13 kDa, and fractions corresponding to g-5′nuc and f-5′nuc were detected at approximately 65 kDa and 17 kDa, respectively. Proteins bands of interest were excised from the gel and submitted to mass spectrometry analysis, which confirmed that the detected bands corresponded to the expected recombinant proteins (Table 2). Protein profiles were analyzed using ImageQuant^TM^ TL software to determine the relative abundance of each purified recombinant protein as previously described [49,50]. The purity of the different recombinant proteins was greater than 90%, and the protein fractions were considered sufficiently pure for ELISA experiments.

**Table 2:** Identification of recombinant salivary proteins by mass spectrometry

**Band number**^**a**^	**Accession number (NCBi)**	**Protein Name**	**Theoretical *MW (kDa)***	**Number of MS/MS peptide sequences**	**Sequence coverage (%)**	**Mascot score**
I	gi|13537666	gSG6 protein [*An. gambiae*]	13.10	9	56.5	1929
II	gi|114864550	gSG6 salivary peptide [*An. funestus*]	13.06	4	25.4	205
III	gi|4582528	putative 5′-nucleotidase [*An. gambiae*]	63.46	29	48.9	3703
IV	gi|114864746	5′ nucleotidase [*An. funestus*]	16.10	6	49.6	819

^a^ The band number corresponds to the same Roman numeral numbers as indicated in Figure 2.

The identities of the bands, their NCBi accession numbers, the theoretical molecular weight values, as well as the number of peptide sequences identified, the corresponding percentage sequence coverage and the Mascot score are listed for MS/MS analysis (Individual ions scores > 16 indicate identity or extensive homology and were considered to be significant (p<0.05).

### IgG response against gSG6 and fSG6 proteins according to anopheline populations and densities

Recently, Rizzo and collaborators evaluated the IgG responses in individuals living in a malaria hyperendemic area of Burkina Faso against recombinant gSG6 and fSG6 proteins from *An. gambiae* and *An. funestus,* respectively [26]. These individuals, exposed predominantly to *An. gambiae s.l.* bites, produced a comparable immune response against both SG6 salivary proteins, indicating a wide cross-reactivity between these anopheline orthologous proteins. The high conservation of the gSG6 sequence between members of the *An. gambiae* species complex (greater than 99%) and members of the *Cellia* subgenus, such as *An. funestus* (with an identity of approximately 80%), supports this idea of shared-antigens [21,25,26]. Based on these results, the gSG6 salivary protein was considered to be a valuable serological marker of exposure to Afrotropical malaria vectors.

However, Rizzo and collaborators did not evaluate the IgG responses against these two orthologous SG6 proteins in individuals exposed predominantly to *An. funestus* mosquito bites. To determine whether variations in the anopheline populations and densities could influence the IgG responses against recombinant orthologs of SG6 proteins from *An. gambiae* and *An. funestus*, the IgG responses against recombinant gSG6 and fSG6 proteins were assessed in our study in 134 individuals living in three Senegalese villages, Diama (n = 46), Ndiop (n = 50) and Dielmo (n = 38) which are considered to be weakly (human biting rate (HBR) < 1) [37-39], moderately (HBR = 3.9) [35,36] and highly (HBR = 30.6) exposed to *Anopheles* bites [34], respectively. These individuals were exposed to distinct anopheline populations, with a predominance of *An. pharoensis* (approximately 87%) and *An. gambiae s.l.* mosquitoes (greater than 94%) in Diama and NDiop, respectively, and a majority of *An. funestus* mosquitoes (66.4%) in Dielmo (Table 1). Sera from European individuals (n = 45) living in Marseille, collected in February 2007 and never exposed to these tropical malaria vectors, were used as un-exposed controls. The IgG response against the gSG6 and fSG6 proteins was assessed by ELISA.

**Figure 3:**
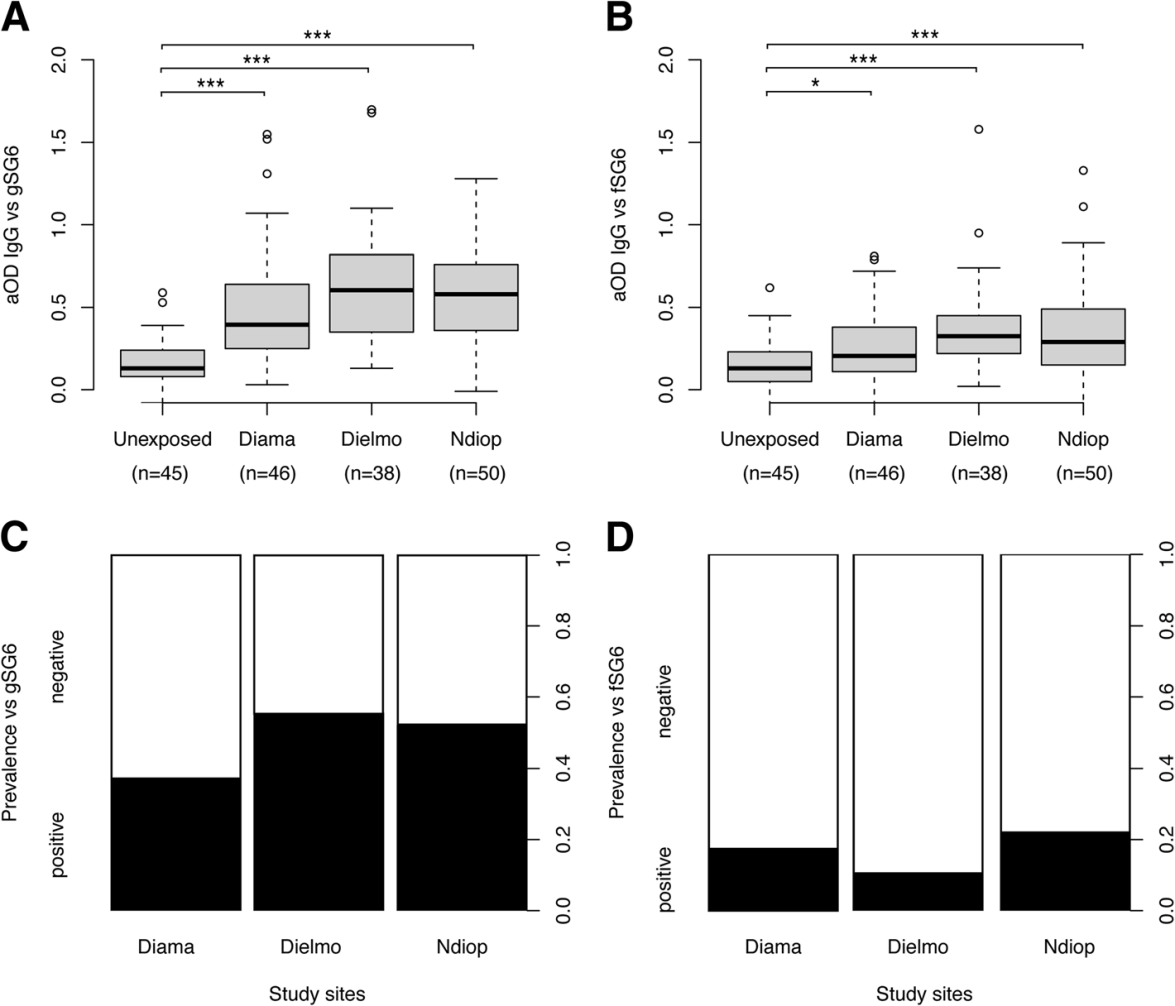
**The IgG response and prevalence to gSG6 and fSG6 according to the level of mosquito bites and *Anopheles *populations (*i.e.,*proportion of mosquito species).** Box plots of aOD values from un-exposed (n = 45) and exposed (Diama, n = 46; Dielmo, n = 38 and Ndiop, n = 50) individuals to gSG6 (**A**) and fSG6 (**B**) proteins. Antibody responses are represented by aOD: the mean OD value of wells with recombinant salivary proteins minus the mean OD value of wells with coating buffer. The box plots display the median aOD value, 25th and 75th percentile. The whiskers indicate the 90th and 10th percentiles and the dots indicate the outliers. The P value was determined according to a Mann–Whitney U test (*, *p <0.05*; **, *p <0.01*; ***, *p <0.001*). The seroprevalence to gSG6 (**C**) and fSG6 (**D**) proteins in the four sites. The cut-off value for seropositivity (the mean aOD ± 3 standard deviations) was defined at 0.55 for gSG6 and 0.59 for fSG6, based on the IgG reactivity of sera from individuals living in Marseille that were not previously exposed to *An. gambiae s.l.* and *An. funestus*. Individuals showing aOD values above the cut-off level for seropositivity were classified as responders. The whiskers denote the 95% CI. The P values were determined by Pearson’s Chi-squared test (*, *p <0.05*; **, *p <0.01*; ***, *p <0.001*).

For gSG6 protein, the IgG responses were significantly different among the four groups (Kruskal-Wallis test, *p<0.0001*, Figure 3A). When comparisons were performed between two sites, the anti-gSG6 IgG responses were significantly higher in exposed individuals from each village (*i.e.,* the means of aODs with a 95% confident interval (95%CI) were +0.50 [0.39 to 0.60], +0.65 [0.53 to 0.76] and +0.58 [0.49 to 0.66] for Diama, Dielmo and NDiop, respectively) compared with un-exposed control individuals (+0.16 [0.12 to 0.20]; Mann–Whitney U test, *p<0.0001*). Conversely, despite distinct exposure levels to *Anopheles* bites in these three Senegalese villages, significant differences were detected only between Diama and Dielmo (Mann–Whitney U test, *p=0.0174*). Although the highest exposure to *Anopheles* bites was in Dielmo, followed by NDiop and finally Diama; the seroprevalence (a seropositivity cut-off was set as the mean aOD of un-exposed controls + 3 SDs: 0.164 + (3 x 0.130) = 0.554), with values of 55%, 52% and 37% in Dielmo, NDiop and Diama, respectively, was not found to be significantly different among the three villages (Pearson’s Chi-squared test, *ns*, Figure 3C). These data support the hypothesis that gSG6 appears to be a valuable marker for distinguishing individuals who are un-exposed to anopheline bites from individuals who have been exposed to anopheline bites, and that this candidate is also well-adapted for the detection low levels of exposure to *Anopheles* bites [20,23]. Although the anti-gSG6 IgG response could discriminate exposed individuals from “low” to “high” *Anopheles* bite densities, such as in Diama and Dielmo, it seems to be less capable of distinguishing “moderate” exposure, such as in NDiop, compared with high and low exposure sites. The individual heterogeneity of the IgG responses against gSG6 in each site could explain this powerless discrimination. This phenomenon could be attributed to the heterogeneity of *Anopheles* exposures according to distinct human behaviors or attractive differences, as recently described [51,52]. To better appreciate the variations of anti-gSG6 IgG levels, according to *Anopheles* densities, a kinetic analysis of the IgG response may be more appropriate and necessary.

For the fSG6 protein, significantly different IgG responses were found among the four groups (Kruskal-Wallis test, *p<0.0001*, Figure 3B). These significant differences were attributed to higher IgG responses from exposed individuals living in Dielmo (*i.e.,* a mean aOD [95%CI] of + 0.38 [0.29 to 0.48], Mann–Whitney U test, *p<0.0001*), NDiop (*i.e.,* + 0.35 [0.26 to 0.44], Mann–Whitney U test, *p<0.0004*) and Diama (*i.e*., + 0.27 [0.19 to 0.34], Mann–Whitney U test, *p=0.0337*) compared with un-exposed control individuals (*i.e.,* + 0.15 [0.10 to 0.19]). As observed for gSG6, a comparison of the IgG responses between two Senegalese villages, indicated a significant difference only between Diama and Dielmo (Mann–Whitney U test, *p=0.0194*). The seroprevalence from Diama (17%), Dielmo (11%) and NDiop (22%) (the seropositivity cut-off was set as the mean aOD of un-exposed controls + 3 SDs: 0.148 + (3 × 0.146) = 0.586) was not found to be significantly different among the three villages (Pearson’s Chi-squared test, *p>0.05*, Figure 3D). As observed for gSG6, the anti-fSG6 IgG responses increased significantly in populations exposed to *Anopheles* bites, even in areas of low exposure; however, this method seems to be insufficiently sensitive to discern different gradients of *Anopheles* densities, and the immune response appears not to be perturbed by the anopheline fauna variations.

### Comparison of the IgG response between SG6 orthologs

**Figure 4:**
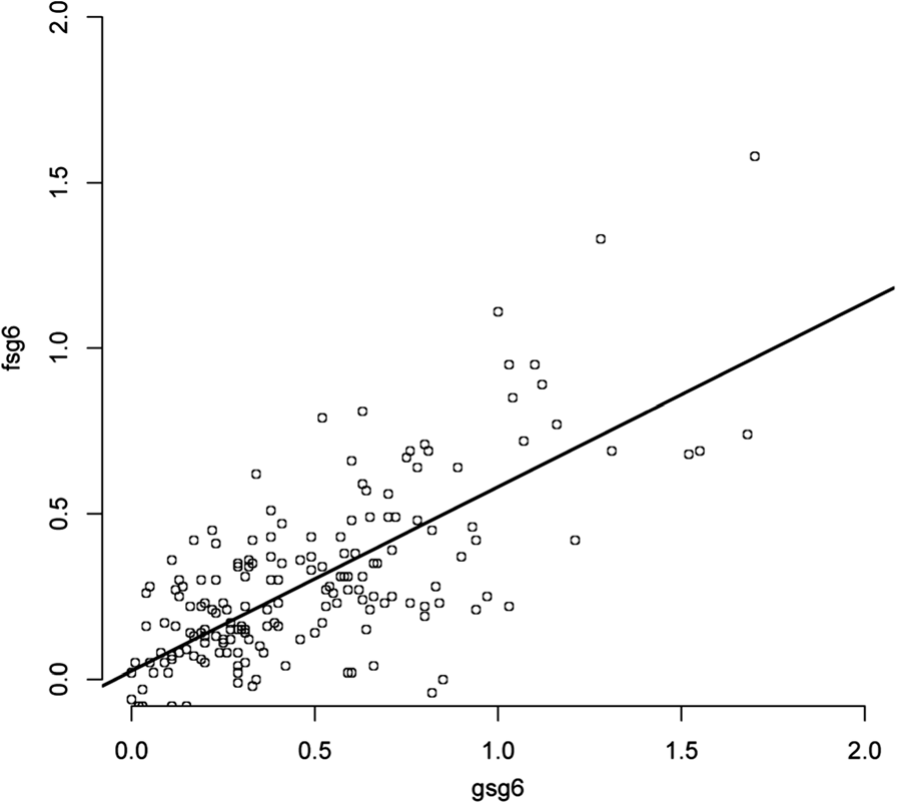
**Correlation of IgG responses between SG6 ortholog recombinant proteins from two distinct *Anopheles *species.** A scatter plot analysis of IgG response to gSG6 is presented, and the aOD values among the 134 exposed individual are reported. For gSG6 and fSG6 measurements, the best-fit is shown as a black line (slope 0.5923±0.0527), with a Spearman’s rank correlation coefficient (rho) of *r* = 0.6208, *p* <0.0001.

To estimate the cross-reactivity level of the IgG responses from exposed individuals of the three Senegalese villages (n = 134) against SG6 orthologs, a Spearman’s rank correlation coefficient (rho) test was used, and the corresponding *p-*values were determined (Figure 4). A significant positive coefficient correlation (r = 0.6208, *p* < 0.0001) was observed between the IgG responses against g-SG6 and f-SG6 among the exposed individuals. Despite the variations of *Anopheles* species proportion among the three villages, the large majority of individuals were “equivalently” distributed around the best-fit line. Thus, these observations strongly support a wide cross-reactivity to the gSG6 and fSG6 salivary antigens, which is in agreement with a previous study [26]. In agreement with Rizzo *et al.*, the slope (0.5923±0.0527) was greater than 0.45, and the best-fit line runs below the diagonal [26]. This phenomenon resulted of in a higher IgG response level against gSG6 compared with fSG6 in the three villages, independent of the *Anopheles* population proportion. Interestingly, the IgG response against the fSG6 protein was significantly lower than that against gSG6, even in areas where individuals are predominantly exposed to *An. funestus* (Wilcoxon signed-rank test, *p<0.0001*, Additional file 4). These results suggest that, despite high sequence conservation among these orthologous SG6 proteins, fSG6 appears to be less antigenic than gSG6. The presence of few species-specific epitopes in each SG6 protein could explain these differences in IgG responses [26].

Collectively, these data uphold that SG6 proteins could be specific markers of exposure to the *Anopheles Cellia*-subgenus, and the use of these two proteins could improve the determination of the individual exposure level to these malaria vectors [26].

### IgG response against g-5′nucleotidase and f-5′nucleotidase proteins according to anopheline populations and density

The presence of numerous *Anopheles* species exhibiting differences in their biology and behavior and variations in mosquito species proportion and density throughout the seasons could have major implications for vector control programs [53,54]. Moreover, the geo-repartition and the density of the *Anopheles* species could vary from area to area in relatively close sites [55], which can increase the complexity of defining the precise regional malaria vectors responsible for malaria transmission. Thus, the achievement of species-specific individual exposure markers from major Afrotropical malaria vectors appears essential to evaluating the appropriateness of vector control measures and to identifying the *Anopheles* species considered to be the main malaria vector in any area at any given time point.

In addition to the SG6 *Anopheline* proteins, this study aimed to evaluate f-5′nuc as a possible *An. funestus* species-specific mosquito-bite-exposure marker. To assess the use of anti-f-5′nuc IgG responses as a tool to distinguish *An*. *funestus* exposure from *An*. *gambiae s.l.* exposure, the immune responses from individuals of the same Senegalese villages were evaluated by an ELISA against 5′nucleotidase ortholog proteins from these two mosquito species.

**Figure 5:**
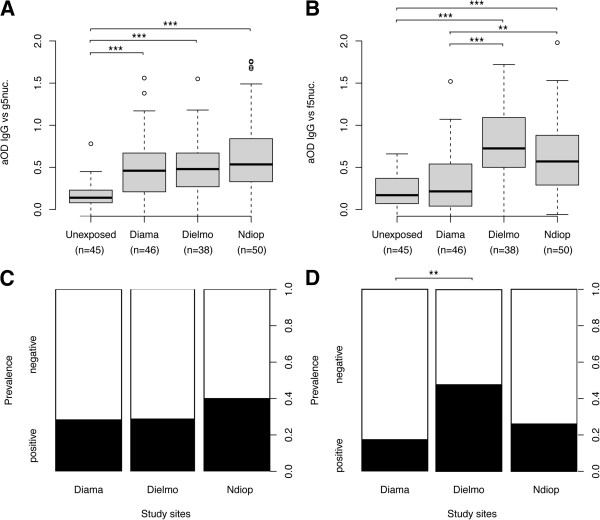
**The IgG response and prevalence to g-5′nuc and f-5′nuc according to the level of mosquito bites and *Anopheles *populations (*i.e.,*proportion of mosquito species).** Box plots of aOD values from unexposed (n = 45) and exposed (Diama, n = 46; Dielmo, n = 38 and Ndiop, n = 50) individuals to g-5′nuc (**A**) and f-5′nuc (**B**) proteins. Antibody responses are represented by aOD: the mean OD value of wells with recombinant salivary proteins minus the mean OD value of wells with coating buffer. The box plots display the median aOD value, 25th and 75th percentile. The whiskers indicate the 90th and 10th percentiles and the dots indicate the outliers. The P value was determined according to a Mann–Whitney U test (*, *p <0.05*; **, *p <0.01*; ***, *p <0.001*). The seroprevalence to g-5′nuc (**C**) and f-5′nuc (**D**) proteins in the four sites. The cut-off value for seropositivity (the mean aOD ± 3 standard deviations) was defined at 0.60 for g-5′nuc and 0.85 f-5′nuc, based on the IgG reactivity of sera from individuals living in Marseille that were not previously exposed to *An. gambiae* and *An. funestus*. Individuals showing aOD values above the cut-off level for seropositivity were classified as responders. The whiskers denote the 95% CI. The P values were determined by Pearson’s Chi-squared test (*, *p <0.05*; **, *p <0.01*; ***, *p <0.001*).

For the g-5′nuc protein, the IgG responses were significantly different among the four groups (Kruskal-Wallis test, *p<0.0001*, Figure 5A). When comparisons were performed between two sites, the anti-g-5′nuc IgG responses were significantly higher in exposed individuals from each village (*i.e.,* the mean aODs [95%CI] were +0.50 [0.40 to 0.61], +0.51 [0.39 to 0.63] and +0.65 [0.52 to 0.77] for Diama, Dielmo and NDiop, respectively) compared with un-exposed control individuals (*i.e.,* +0.16 [0.12 to 0.21]; Mann–Whitney U test, *p<0.0001*). Conversely, no significant differences in the anti-g-5′nuc IgG responses was found, regardless of which two groups from these three Senegalese villages were being compared (Mann–Whitney U test, *ns*). Although a higher seroprevalence (the seropositivity cut-off was set as the mean aOD of un-exposed controls + 3 SDs: 0.163 + (3 x 0.147) = 0.604) was detected among individuals living in the NDiop area (40%), where *An. gambiae s.l.* is largely predominant, compared with Diama (28%) and Dielmo (29%), no significant differences were found among the three villages (Pearson’s Chi-squared test, *ns*, Figure 5C). These data suggest that the anti-g-5′nuc IgG responses could distinguish individuals un-exposed to *Anopheles* from individuals exposed to *Anopheles*, independent of the level of exposure or the anopheline fauna.

For the f-5′nuc protein, the IgG responses were significantly different among the four groups (Kruskal-Wallis test, *p<0.0001*, Figure 5B). In particular, the anti-f-5′nuc IgG responses were significantly higher for exposed individuals living in Dielmo (*i.e.,* + 0.80 [0.65 to 0.96]) and NDiop (*i.e.,* + 0.63 [0.50 to 0.76]), compared with un-exposed control individuals (Mann–Whitney U test, *p<0.0001* for these two comparisons) or with individuals living in Diama (Mann–Whitney U test, *p<0.0001* compared with Dielmo and *p<0.002* compared with NDiop). No significant difference was noted between individuals living in Diama (*i.e.,* a mean aOD [95%CI] of + 0.34 [0.22 to 0.46]) and un-exposed control individuals (*i.e.,* + 0.22 [0.16 to 0.28]; Mann–Whitney U test, *ns*). The seroprevalence (the seropositivity cut-off was set as the mean aOD of un-exposed controls + 3 SDs: 0.219 + (3 x 0.210) = 0.849) was found to be significantly different among individuals living in Dielmo (47%) and those living in Diama (17%) (Pearson’s Chi-squared test, *p<0.007*, Figure 5D), corresponding to areas where *An. funestus* is predominantly present and absent, respectively. The seroprevalence of individuals living in NDiop (26%) was not significantly different when compared with the two other Senegalese villages (Pearson’s Chi-squared test, *ns*).

The absence of a significant anti-f-5′nuc IgG response among un-exposed individuals and among individuals living in Diama that were not exposed to *An. funestus* bites, in addition to the detection of highly significant differences in the anti-f-5′nuc IgG responses between individuals not exposed to *An. funestus* bites and individuals living in Dielmo or NDiop, suggests that exposure to *An. funestus* bites seems to be required to elicit an antibody response against the f-5′nuc protein. Indeed, the higher IgG response against f-5′nuc that was detected at a site where *An. funestus* is predominant is a supplementary argument supporting the idea that this salivary protein could reflect a species-specific exposure. Nevertheless, the data could not exclude the possibility that the anti-f-5′nuc IgG responses could correspond to the level of *Anopheles* bites. Indeed, the anopheline specimens collected in these three villages suggested a decreasing mosquito density gradient from Dielmo to Diama, with an intermediate exposure level in NDiop, which corresponds to the anti-f-5′nuc IgG pattern observed among these three areas. Comparisons of the anti-f-5′nuc IgG responses from individuals exposed uniquely to *An. funestus* or to *An. gambiae s.l.* at equivalent densities could dispel this ambiguity. Nevertheless, paired comparisons of the IgG responses against 5′nuc orthologous proteins indicated a significant decrease and increase for individuals living in Diama (Wilcoxon signed-rank test, *p=0.023*) and Dielmo (Wilcoxon signed-rank test, *p=0.004*), respectively (Additional file 4).These data suggest that the recognition of these salivary proteins by the immune system was not equivalent and that specific antigenic epitopes may be associated with each protein.

### Comparison of the IgG response between 5′nucleotidase orthologs

**Figure 6:**
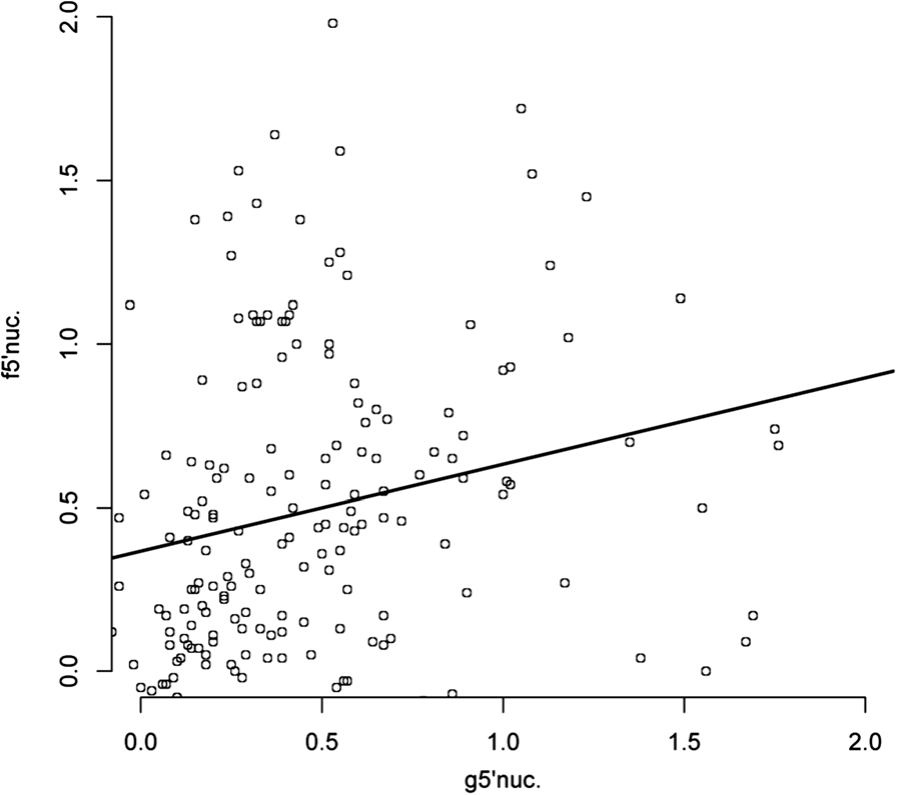
**Correlation of IgG responses between 5′nucleotidase ortholog recombinant proteins from two distinct *Anopheles *species.** A scatter plot analysis of IgG response to f-5′nuc is presented, and the aOD values among the 134 exposed individual are reported. For g-5′nuc and f-5′nuc measurements, the best-fit is shown as a black line (slope 0.1245±0.1036), with a Spearman’s rank correlation coefficient (rho) of *r* = 0.1635, *p*>0.05 (*p*=0.0591).

To assess the cross-reactivity level of the IgG response of exposed individuals from the three Senegalese villages (n = 134) against 5′nuc orthologs, a Spearman’s rank correlation coefficient (rho) test was used, and the corresponding *p-*values were determined. In contrast to the SG6 orthologs, no significant correlation was observed between the IgG responses from exposed individuals against g-5′nuc and f-5′nuc recombinant proteins (r = 0.1635, *p*=0.0591). A high dispersion of the dots was visible on the scatter plot (Figure 6), underlining the fact that some individuals presented a larger response to either g-5′nuc or to f-5′nuc proteins. Thus, the antibody response against these 5′nuc ortholog proteins indicated a low cross-reactivity, suggesting that, despite a partial conservation of the carboxy-terminal end of these proteins (74% identical), the two proteins may be harboring “species”-specific epitopes. In addition, the large amino-terminal segment of g-5′nuc (*i.e.,* from amino acid 1 to 434) could possibly possess antigenic sequences that are absent in f-5′nuc and could explain the differences in serological recognition. Moreover, the g-5′nuc amino-terminal sequence possesses several cysteines (n = 7) that may be involved in protein folding, which could change the epitope conformation or mask some epitopes in the carboxy-terminal (which has partial homology with f-5′nuc protein sequence). Indeed, the resulting cryptic epitopes in the g-5′nuc protein could be, in contrast, accessible to an antibody response in the f-5′nuc protein and, therefore, induce a “species”-specific serological response.

## Conclusion

Understanding the complexity of the *Anopheles* species behavior is of major importance in vector control interventions to protect human populations against malaria. *Anopheles gambiae s.l.* and *An. funestus* share particularly anthropophilic tendencies that contribute to their vectorial capacity [56]. However, the existence of ecological and behavioral differences between these species have important epidemiological consequences [57]. Indeed, during the dry season, the densities of *An. gambiae s.l.* declined in some Sub-Saharan Africa areas, whereas the *An. funestus* abundance remained maximal, extending the period of malaria transmission. Therefore, the determination of human exposure to malaria vectors at the quantitative level (*e.g.,* mosquito bite densities) and at the qualitative level (*e.g.,* which mosquito species bite humans) should help to adapt malaria control strategies according to the spatial and temporal density of mosquito fauna [58].

To this end, the analysis of the human antibody response against mosquito salivary antigens has proved to be a relevant tool to assess host/vector contact [16,17,59]. As some areas can exhibit a high biodiversity in terms of mosquito species, a high level of specificity is necessary to assess individual exposure by immunological tests based on mosquito saliva. In addition, the presence of a diverse degree of salivary antigen cross-reactivity between different vector species demonstrates the need to precisely define antigenic candidate biomarkers to reflect exposure to several *Anopheles* species and to distinguish vector exposure at the species level [9].

Therefore, the production of specific mosquito saliva antigens in a recombinant form or by using synthetic peptides is a promising alternative strategy for producing safe and highly standardized antigens on a large scale [21,24]. A gain of specificity could be achieved by the use of synthetic peptides that do not share sequence homology with other hematophagous arthropod species [40]. However, the production of whole recombinant antigenic proteins could be more efficient to detect mosquito exposure [25].

The well-conservedSG6 protein family within the *Anopheles Cellia* subgenus made the SG6 proteins the first anopheline salivary candidates tested for the exploration of the relationship between levels of anti-gSG6 IgG responses and individual exposure to *Anopheles* bites. Encouraging data has accumulated for the use of the gSG6 salivary protein as serological marker of exposure to the *Anopheles* genus. Incontestably, the gSG6 protein can be used to detect exposure to *Anopheles* bites, even in areas of low exposure [23]. In the present study, the IgG response against SG6 from *An. gambiae* and *An. funestus* could distinguish individuals un-exposed to *Anopheles* bites from individuals exposed to *Anopheles* bites and could also distinguish high levels of exposures, independent of the anopheline *Cellia* species fauna. However, gSG6 was found to elicit a higher level of response than fSG6 orthologs. Overall, the analysis of IgG responses against SG6 ortholog proteins sustains the use of SG6 proteins as consistent indicators of exposure to three major malaria vectors in tropical Africa (*i.e., An. gamgiae, An. arabiensis* and *An. funestus*).

In contrast, the presence of significantly different patterns and intensities of IgG responses against 5′nucleotidase anopheline orthologs supports the idea that each of these proteins should possess specific antigenic epitopes. Moreover, the IgG response level against the f-5′nuc protein seems to be associated with *An. funestus* densities. These initial tests provided encouraging preliminary information on the immunogenicity of the anopheline 5′nuc proteins and present the promising possibility of using the 5′nucleotidase salivary protein from *An. funestus* as the first species-specific antigenic marker of exposure. Complementary studies are needed to confirm the present assumption.

## Abbreviations

ACN: Acetonitrile; aOD: adjusted optical density; CI: Confident interval; CV: co-efficient of variation; ELISA: Enzyme-Linked ImmunoSorbent Assays; HBR: Human biting rate; MS: Mass spectrometry; NCBI: National Center for Biotechnology Information; SD: Standard deviation; SG6: Salivary gland protein 6.

## Competing interests

The authors declare that they have no competing interests.

## Authors’ contributions

AL and RC conceived and designed the experiments. ZA and BM performed the experiments. AL, FA, RC, FC and PF analysed the data. BN, AS, BY, RF, VHV contributed reagents/materials/analysis tools. AL, FC, FA and RC wrote the paper. All authors read and approved the final manuscript.

## Supplementary Material

Additional file 1 **Paired-wise alignment of 5′-nucleotidase proteins from *An. gambiae *and *An.funestus.*** Click here for file

Additional file 2 **Comparison of sequence alignment of *Culicidae* protein members from the 5′ nucleotidase/Apyrase family to 5′-nucleotidase proteins from *An. gambiae *(gi|4582528).** Click here for file

Additional file 3 **Phylogram tree constructed from the alignment of the SG6 protein sequences from *Anopheles* species.** Click here for file

Additional file 4 Statistical analysis of variations in IgG responses per site against the anopheline salivary proteins. Click here for file
