# Supplementary figures and images for: Assessment of Anopheles salivary antigens as individual exposure biomarkers to species-specific malaria vector bites
Source: Malar J. 2012 Dec 31;11:439. doi: 10.1186/1475-2875-11-439 (PMC3547717; doi:10.1186/1475-2875-11-439)

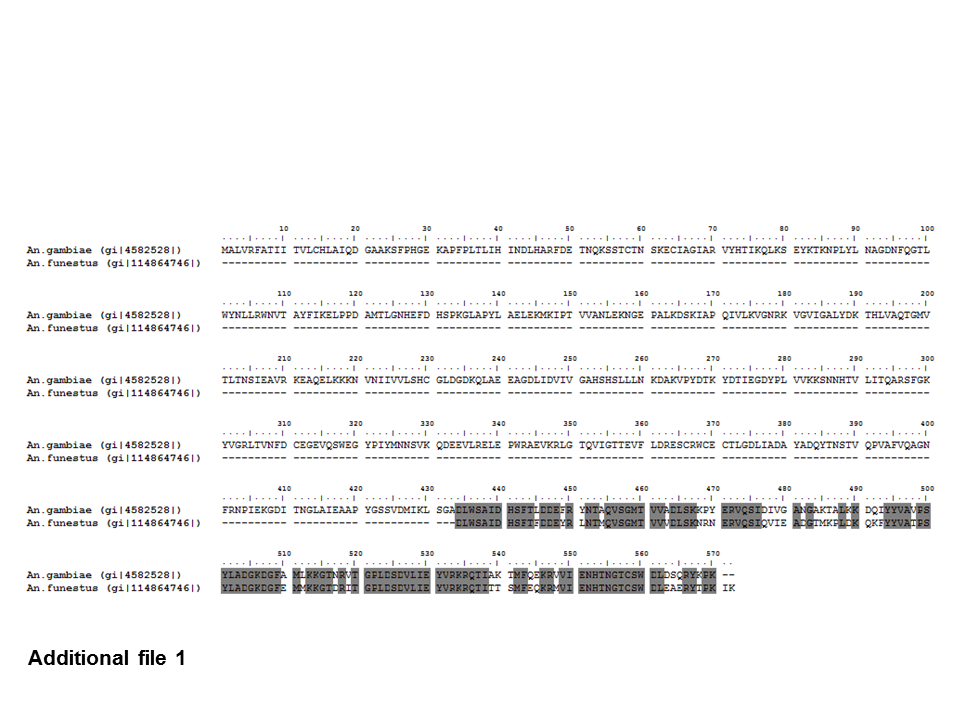

Supplement: Additional file 1 — Paired-wise alignment of 5′-nucleotidase proteins from An. gambiae and An.funestus. [file 1475-2875-11-439-S1.tiff]

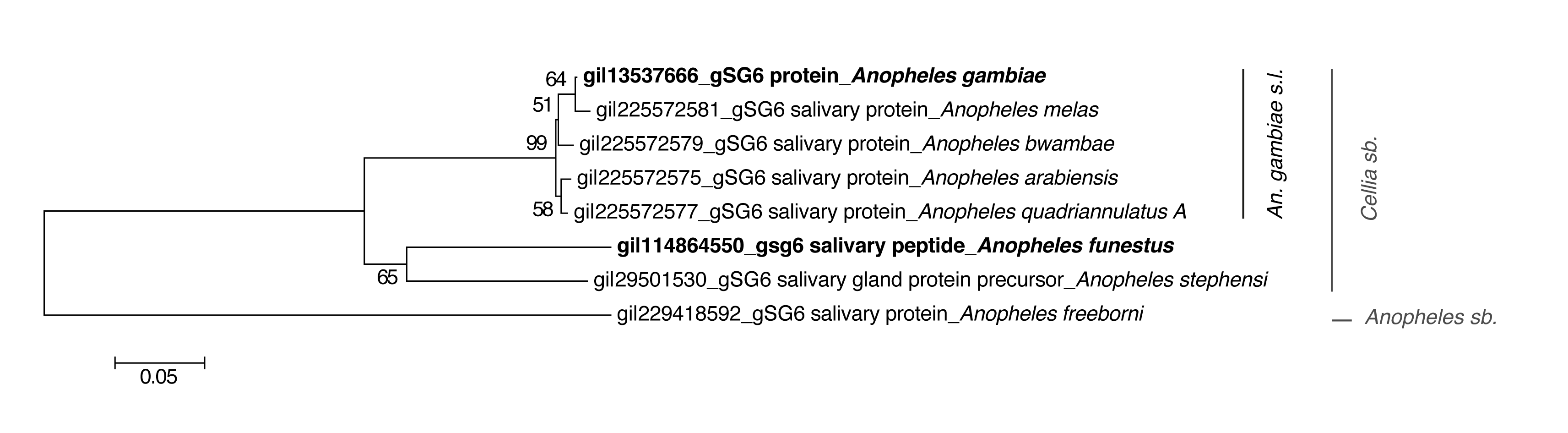

Supplement: Additional file 3 — Phylogram tree constructed from the alignment of the SG6 protein sequences from Anopheles species. [file 1475-2875-11-439-S3.tiff]
