# Supplementary material for: Assessment of Anopheles salivary antigens as individual exposure biomarkers to species-specific malaria vector bites
Source: Malar J. 2012 Dec 31;11:439. doi: 10.1186/1475-2875-11-439 (PMC3547717; doi:10.1186/1475-2875-11-439)
Supplement: Additional file 2 — Comparison of sequence alignment of Culicidae protein members from the 5′ nucleotidase/Apyrase family to 5′-nucleotidase proteins from An. gambiae (gi|4582528). [file 1475-2875-11-439-S2.doc]

**Additional File 2**. Comparison of sequence alignment of Culicidae protein members from the 5’ nucleotidase/Apyrase family to 5’-nucleotidase proteins from *An. gambiae* (gi|4582528).

| **Accession number** (NCBi) | **Protein Name** | **Species** | **Identity** | **Coverage** |
| --- | --- | --- | --- | --- |
| gi|4582528 | putative 5'-nucleotidase | *Anopheles gambiae* | 100% | 100% |
| gi|58377530 | AGAP011026-PA | *Anopheles gambiae str. PEST* | 99% | 100% |
| gi|27372911 | salivary apyrase | *Anopheles stephensi* | 80% | 98% |
| gi|208657633 | putative 5' nucleotidase/apyrase | *Anopheles darlingi* | 66% | 100% |
| gi|556272 | apyrase | *Aedes aegypti* | 59% | 93% |
| gi|763502 | apyrase | *Aedes aegypti* | 59% | 93% |
| gi|312379566 | hypothetical protein AND_08534 | *Anopheles darlingi* | 50% | 90% |
| gi|56417436 | salivary apyrase | *Aedes albopictus* | 60% | 90% |
| gi|374110470 | salivary apyrase | *Aedes albopictus* | 60% | 90% |
| gi|312379568 | hypothetical protein AND_08536 | *Anopheles darlingi* | 60% | 89% |
| gi|157113141 | apyrase, putative Aedes aegypti | *Aedes aegypti* | 62% | 88% |
| gi|170049736 | apyrase | *Culex quinquefasciatus* | 57% | 88% |
| gi|170049743 | apyrase | *Culex quinquefasciatus* | 57% | 85% |
