# Supplementary material for: Assessment of Anopheles salivary antigens as individual exposure biomarkers to species-specific malaria vector bites
Source: Malar J. 2012 Dec 31;11:439. doi: 10.1186/1475-2875-11-439 (PMC3547717; doi:10.1186/1475-2875-11-439)
Supplement: Additional file 4 — Statistical analysis of variations in IgG responses per site against the anopheline salivary proteins. [file 1475-2875-11-439-S4.doc]

**Additional file 4.** Statistical analysis of variations in IgG responses per site against the anophelines salivary proteins.

|  |  |  |  | 95% CI | | *p-value*  (Wilcoxon signed-rank tests) | | | *p-value* |
| --- | --- | --- | --- | --- | --- | --- | --- | --- | --- |
|  | Time | Mean | SD | Lower | Upper | gSG6 vs. | fSG6 vs. | g5’nuc vs. | (Friedman test) |
| **Unexposed (aOD)** |  |  |  |  |  |  |  |  |  |
|  | gSG6 | 0.164 | 0.134 | 0.124 | 0.205 |  |  |  | 0.082 |
|  | fSG6 | 0.148 | 0.146 | 0.104 | 0.192 |  |  |  |
|  | g5’nuc | 0.163 | 0.147 | 0.119 | 0.207 |  |  |  |
|  | f5’nuc | 0.219 | 0.210 | 0.156 | 0.282 |  |  |  |
| Mean |  | 0.174 |  |  |  |  |  |  |  |
| SD |  | 0.031 |  |  |  |  |  |  |  |
| **Diama (aOD)** |  |  |  |  |  |  |  |  |  |
|  | gSG6 | 0.496 | 0.358 | 0.390 | 0.602 |  |  |  | **2.2e-16** |
|  | fSG6 | 0.266 | 0.257 | 0.190 | 0.343 | **2.49e-06** |  |  |
|  | g5’nuc | 0.504 | 0.366 | 0.396 | 0.613 | 0.682 | **2.922e-05** |  |
|  | f5’nuc | 0.340 | 0.402 | 0.221 | 0.459 | **0.018** | 0.328 | **0.022** |
| Mean |  | 0.401 |  |  |  |  |  |  |  |
| SD |  | 0.118 |  |  |  |  |  |  |  |
| **Dielmo (aOD)** |  |  |  |  |  |  |  |  |  |
|  | gSG6 | 0.647 | 0.354 | 0.530 | 0.763 |  |  |  | **2.2e-16** |
|  | fSG6 | 0.383 | 0.283 | 0.290 | 0.476 | **4.681e-07** |  |  |
|  | g5’nuc | 0.512 | 0.368 | 0.391 | 0.633 | **0.017** | *0.055* |  |
|  | f5’nuc | 0.803 | 0.466 | 0.650 | 0.957 | **0.038** | **4.425e-06** | **0.004** |
| Mean |  | 0.586 |  |  |  |  |  |  |  |
| SD |  | 0.180 |  |  |  |  |  |  |  |
| **NDiop (aOD)** |  |  |  |  |  |  |  |  |  |
|  | gSG6 | 0.575 | 0.301 | 0.490 | 0.661 |  |  |  | **0.001** |
|  | fSG6 | 0.340 | 0.309 | 0.262 | 0.437 | **1.47e-07** |  |  |
|  | g5’nuc | 0.646 | 0.446 | 0.519 | 0.773 | 0.828 | **3.38e-05** |  |
|  | f5’nuc | 0.632 | 0.466 | 0.500 | 0.765 | 0.769 | **3.1e-04** | 0.695 |
| Mean |  | 0.548 |  |  |  |  |  |  |  |
| SD |  | 0.142 |  |  |  |  |  |  |  |
|  |  |  |  |  |  |  |  |  |  |

The Friedman tests were used to compare the antibody levels between more than two salivary proteins. Wilcoxon signed-rank tests were used to compare sera from the same site between two salivary proteins. All significant differences (*p*<0.05) are indicated in bold. SD: standard deviation, aOD: adjusted optical density, CI: confident interval.
